# Supplementary material for: Asymmetry of carbon sequestrations by plant and soil after forestation regulated by soil nitrogen
Source: Nat Commun. 2023 Jun 2;14:3196. doi: 10.1038/s41467-023-38911-w (PMC10238465; doi:10.1038/s41467-023-38911-w)
Supplement: Supplementary file 1 — Supplementary Information [file 41467_2023_38911_MOESM1_ESM.pdf]

Asymmetry of carbon sequestrations by plant and soil after forestation regulated by soil nitrogen

Supplementary information

Songbai Hong[1], Jinzhi Ding[2*], Fei Kan[1], Hao Xu[1],

Shaoyuan Chen[1], Yitong Yao[3], Shilong Piao[1, 2*]

[1] Institute of Carbon Neutrality, Sino-French Institute for Earth System Science, College of Urban and Environmental Sciences, Peking University, Beijing 100871, China.

[2] State Key Laboratory of Tibetan Plateau Earth System, Resources and Environment, Institute of Tibetan Plateau Research, Chinese Academy of Sciences, Beijing 100101, China.

[3] Division of Geological and Planetary Sciences, California Institute of Technology, Pasadena, California 91125, USA.

Supplementary Figures

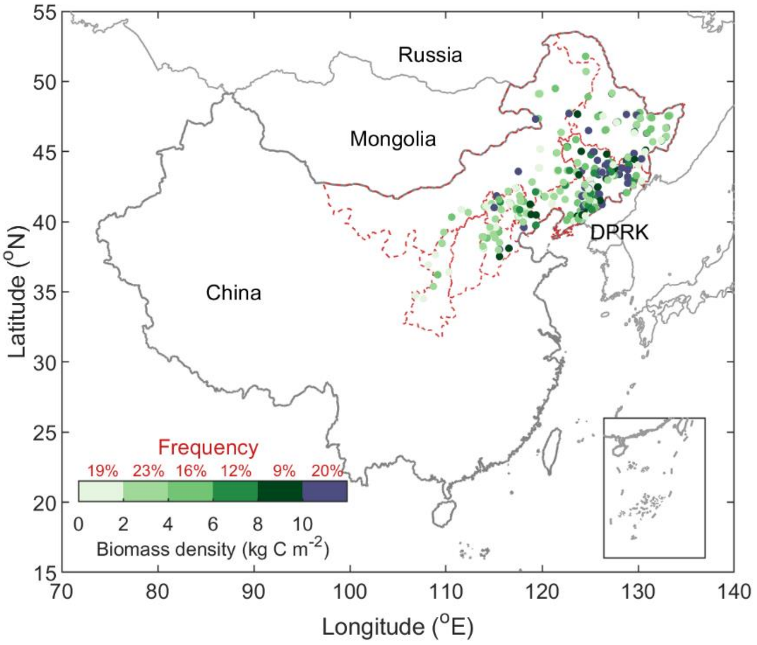

Supplementary Figure 1. The study area and the spatial distribution of biomass density in planted forest plots. The percentages above the color bar indicate the relative frequency of data in each interval. The base map was derived without endorsement from GADM data (https://gadm.org/), and the map was generated in MATLAB R2020a (MathWorks).

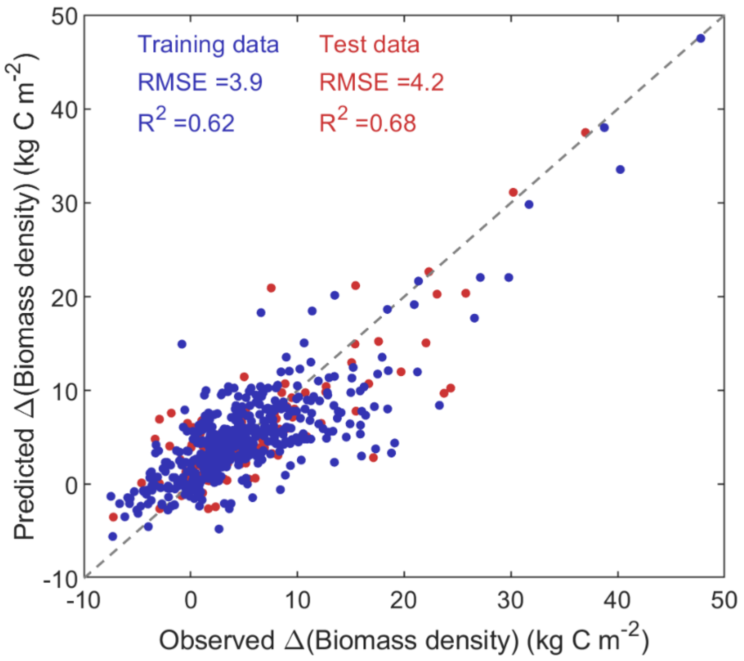

Supplementary Figure 2. The performance of model tree ensemble (MTE) in estimating changes in biomass density induced by forestation. Mean annual precipitation (MAP), mean annual temperature (MAT), tree species, stand age, longitude, and latitude were used as the predictors in the MTE.

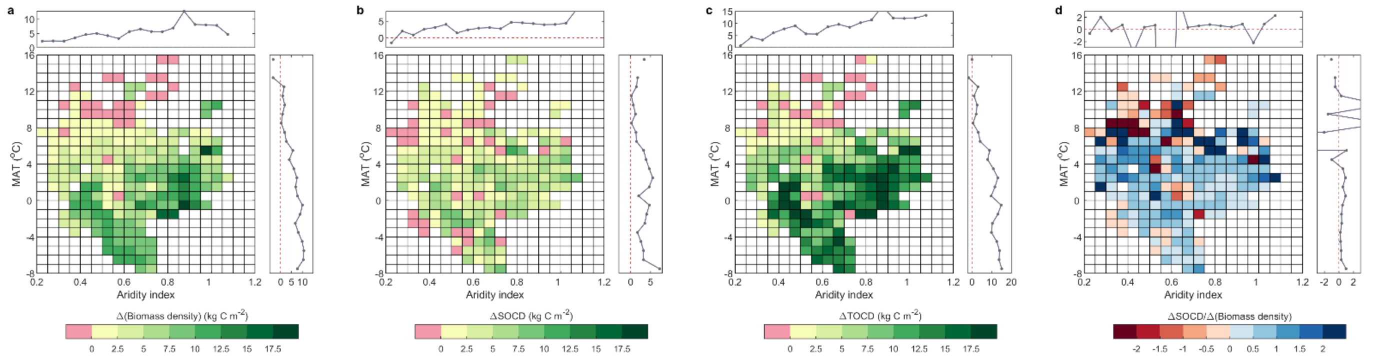

Supplementary Figure 3. Divergent responses of biomass and soil organic carbon (SOC) densities to forestation (Δ(biomass density) and ΔSOCD) along climate gradients. **a-d**. The distribution of Δ(biomass density), ΔSOCD, ΔTOCD (i.e. Δ(biomass density)+ΔSOCD), and ΔSOCD/Δ(biomass density) in a two-dimension space of mean annual temperature (MAT) and aridity index (AI). The mean values for each interval, derived from the output of machine learning, are shown. The top line chart in panel **a** indicates the variation of Δ(biomass density) along the AI gradient, while the chart on the right-hand side indicates the variation of Δ(biomass density) with MAT. Mean values for each interval were used to generate the lines. The line charts in panel **b-d** were created similarly.

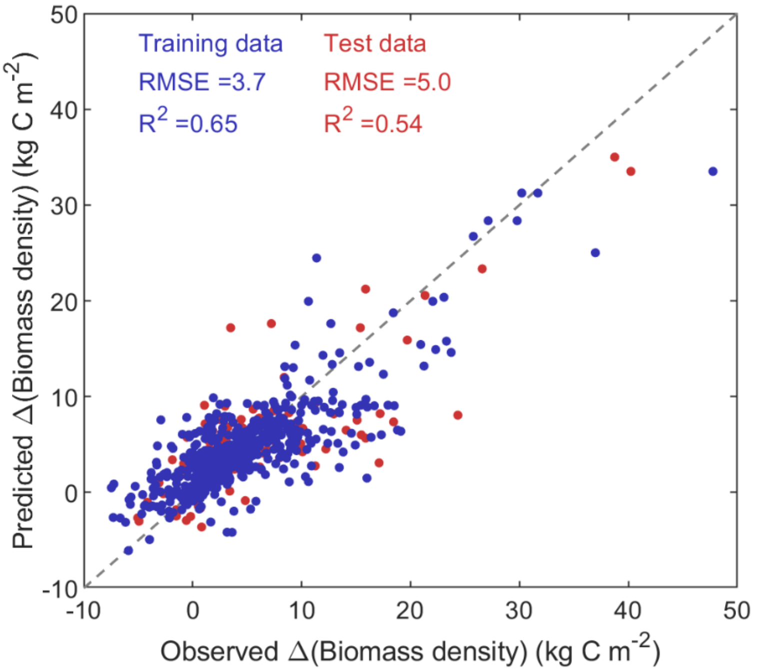

Supplementary Figure 4. The performance of model tree ensemble (MTE) in estimating changes in biomass density induced by forestation. The original vegetation and land use type, mean annual precipitation (MAP), mean annual temperature (MAT), tree species, stand age, longitude, and latitude were used as the predictors in the MTE.

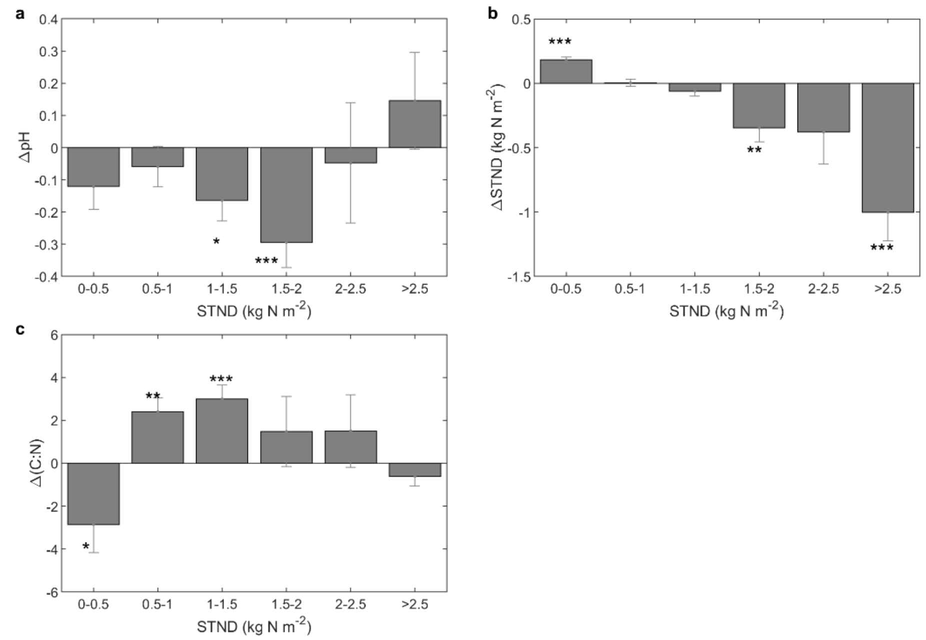

Supplementary Figure 5. Changes in soil pH, soil total nitrogen density (STND) and soil C:N induced by forestation along background STN gradient. a-c indicate changes in soil pH, STND and soil C:N, respectively. Independent sample *t*-tests with correction for false discovery rates were conducted to compare the data of each group with 0. *, ** and *** indicate that the null hypothesis can be rejected at $p < 0.05$, 0.01 and 0.001, respectively. Error bars indicate standard errors.

Supplementary tables

Supplementary Table 1. Changes in biomass density (kg C m^{-2}) induced by forestation with different tree species in different original vegetation and land use type. The mean values, standard errors and the sample size (in brackets) are shown in the table. Numbers in bold indicate the changes are significantly different from 0 ($p < 0.05$) based on independent sample *t*-tests.

	P. koraiensi	*L. gmelinii*	*P. sylvestris* var. *mongholica*	*P. tabuliformis*	*Populus* spp.	*Overall*
Cropland	**5.07±0.47** (14)	**4.44±0.13** (72)	**7.26±0.54** (15)	3.25±0.80 (5)	**6.00±0.06** (70)	**5.31±0.04** (176)
Barren land	**4.09±0.18** (19)	**5.68±0.06** (118)	**7.20±0.24** (36)	**1.26±0.03** (67)	**3.74±0.06** (53)	**4.41±0.02** (293)
Grassland		1.57±0.49 (8)	**3.96±0.37** (6)	1.24 (1)	**2.46±0.38** (7)	**2.49±0.14** (22)
Natural forest	**4.20±0.17** (22)	**6.46±0.14** (66)	7.07±2.02 (4)	1.38±0.36 (10)	4.38±1.33 (3)	**5.47±0.07** (105)
Riparian sand land					**5.24±0.15** (18)	**5.24±0.15** (18)
Total	**4.38±0.08** (55)	**5.41±0.03** (264)	**6.89±0.13** (61)	**1.40±0.03** (83)	**4.92±0.03** (151)	**4.80±0.01** (614)

Supplementary Table 2. The plantation areas (km^2) of each tree species in each province. Data derived from the State Forestry Administration of the People's Republic of China, Eighth National Forest Resource Inventory Report (2009–2013) [ref 1].

	Pinus koraiensis	*Larix gmelinii*	*Pinus sylvestris* var. *mongolica*	*Pinus tabuliformis*	*Populus* spp.	Others	Total
Heilongjiang	1724	10752	1945		6526	3132	24079
Jilin	494	5737	788	43	4783	3401	15246
Liaoning	506	4077	349	4041	3855	5501	18329
Hebei		2655	64	2413	4977	3631	13740
Shanxi		821	32	2526	2101	2611	8091
Shaanxi		96		2495	1021	8250	11862
Inner Mongolia		5600	990	2374	19978	2705	31647
Total	2724	29738	4168	13892	43241	29231	122994

Supplementary Table 3. Coefficients for calculating timber volume (Based on ref [2-6]).

	a	e	f
P. koraiensi	0.0000589865	1.966609091	0.904763956
L. gmelinii	0.00005779	1.76845554	1.10597809
Populus spp.	0.000071	1.576540	1.193517
P. tabuliformis	0. 000196669	1.79887	0.5118834
P. sylvestris var. *mongholica*	0.0000966	1.8146	0.8276

Supplementary Table 4. Coefficients for converting timber volume to biomass density. (Based on ref [7]).

	b	c
P. koraiensi	0.5185	18.22
L. gmelinii	0.6096	33.806
Populus spp.	0.4754	30.6034
P. tabuliformis	0. 7554	5.0928
P. sylvestris var. *mongholica*	1.0945	2.0040

Supplementary references

1. State Forestry Administration of the People's Republic of China, Eighth National Forest Resource Inventory Report (2009–2013), (2014).
2. Fang, C. et al. Construction of bivariate stem volume tables of poplar in Chaoyang region. Journal of Liaoning Forestry Science & Technology, 4, 5-7 (2001a).
3. Gong, S. & Liu, D. Establishment of the Tree Volume Tables of *Pinus Sylvestris* var *mongolica* Plantation in Daxinganling Region. Forest Resources Management, 5 (2002).
4. Song, Y. Establishment of binary tree volume table of Larix gmellini (Rupr.) Rupr plantation. Forestry Prospect and Design, 1, (2012).
5. Wang, Y. & Wu, F. Study on the compilation of timber volume table of *Pinus tabulaeformis* plantation in Fuxin. Journal of Northeast Forestry University, 15, 5 (1987).
6. Yang, H. et al. Reformulation of binary timber volume table of artificial Korean pine in Liaoning Province. Journal of Liaoning Forestry Science & Technology, 2, 61 (2007).
7. Fang, J., Chen, A., Peng, C., Zhao, S. & Ci, L. Changes in Forest Biomass Carbon Storage in China Between 1949 and 1998. Science 292, 2320-2322 (2001b).
